# Contribution of preoperative ultrasound-guided implantation of a magnetic seed for optimal localization and resection of vulvar angiosarcoma: A case report

**DOI:** 10.1016/j.ijscr.2023.109107

**Published:** 2023-12-08

**Authors:** Pauline Chapellier, Basile Pache, Laura Haefliger, Loïc Lelièvre, Patrice Mathevet, Rami Hajri

**Affiliations:** a Department of Radiology, Lausanne University Hospital (CHUV), Lausanne, Switzerland; b Department Women-Mother-Child, Gynecology and Obstetrics Unit, Lausanne University Hospital (CHUV), Lausanne, Switzerland; c University of Lausanne (UNIL), Lausanne, Switzerland

**Keywords:** Case report, Vulvar neoplasm, Ultrasound, Imaged-guide surgery, Localization

## Abstract

**Introduction:**

Vulvar cancer is a rare cause of malignancy among women. It is key for surgeons to achieve negative resection margins, as it greatly impacts patient's prognosis. Unfortunately, additional surgical procedures are often performed due to the regional anatomical complexity.

Based on non-palpable breast tumors, where image-guided preoperative localization tools have enhanced the complete resection rates, we aimed at evaluating the feasibility of magnetic seed technique for localizing perineal lesions.

**Presentation of the case:**

We present the case of a 40-year-old female patient, who underwent iterative resections for a recurrent epithelioid angiosarcoma of the left labia major. Imaging revealed a suspicious regional involvement at 3 months of follow-up, for which another surgery was planned.

We decided to target this non-palpable lesion with the Magnetic Seed technique to guide the intervention. A seed was inserted into the nodule under ultrasound guidance. Resection was then performed, with negative margins and no recurrence on last follow-up.

**Discussion:**

Surgical procedures with minimal extension are recommended in vulvar cancer, to limit the aesthetic and functional complication. Unfortunately, recurrences and residual tumors remain frequent, even higher when surgical margin safety is not achieved.

Many studies have suggested the benefit of image-guided localization tools in non-palpable breast tumors. By reducing the excising volume and focusing on the lesions, relapse and complications are rarer. We considered Magnetic Seed to be the most appropriated technique for perineal lesions.

**Conclusion:**

As for breast cancer, Magnetic Seed technique could be appropriate for non-palpable perineal lesions, optimizing resection margins with minimal procedures.

## Highlights

• Focal vulvar cancer surgery tends to limit aesthetic and functional complications.
• Vulvar cancer presents a high overall incidence of local recurrence after surgery.
• Pre-operative localization with Magnetic Seed can be performed for perineal lesions.

## 1 Introduction

Vulvar cancer is an uncommon gynecological malignancy, representing around 5 % of all female genital tract tumors [1]. With a median diagnosis age of 65, it primarily affects postmenopausal women, although the incidence is increasing among younger women [2].

Depending on the stage and characteristics of the tumor, the management of vulvar cancer frequently involves a multidisciplinary approach, including surgery, radiation therapy, and chemotherapy. In case of localized forms, surgery in combination with radiotherapy has become the standard treatment, ranging from tumor resection to radical vulvectomy [3].

Precise localization of tumor for its complete removal is crucial in patient's journey to recovery.

However, studies suggest a high overall incidence of local recurrence in vulvar carcinoma [4], mostly due to positive margins. Therefore, adjuvant radiotherapy is often planned, leading to soft tissue changes and difficulties to perform a clinical assessment. Advanced imaging is often used for the follow-up and early detection of recurrence (PET/CT + MRI).

In this multidisciplinary approach, the development of new technologies enhancing tumor preoperative localization has evolved.

In case of non-palpable tumors, image-guided preoperative localization tools allow the surgeon to correctly localize the tumor and perform an accurate resection. These tools include wire-guide localization, radio-guided localization, magnetic tracers, and magnetic seed localization [5,6]. The use of magnetic seeds is a new promising technique, avoiding any radiation, and facilitating the coordination between radiologists and surgeons. It consists in implanting under ultrasound guidance a stainless steel magnetic seed, which is in a second step detected during surgery thanks to a handheld magnetic probe, with an equivalent or increasing rates of complete resection margins compared to the other methods, depending on studies [[7], [8], [9], [10]]. It reduces the risk of complications such as potential displacement and local discomfort for the patients, alongside with the advantage of not requiring surgery immediately after localization as the seed remains stable in time and space [5].

This procedure has been described in breast oncosurgery [11,12] but has not been described in other locations yet. The aim of this case report is to present this technique applied to an unpalpable vulvar angiosarcoma recurrence. This publication has been reported in line with the SCARE criteria [13].

## 2 Case report/series

**Fig. 1:**
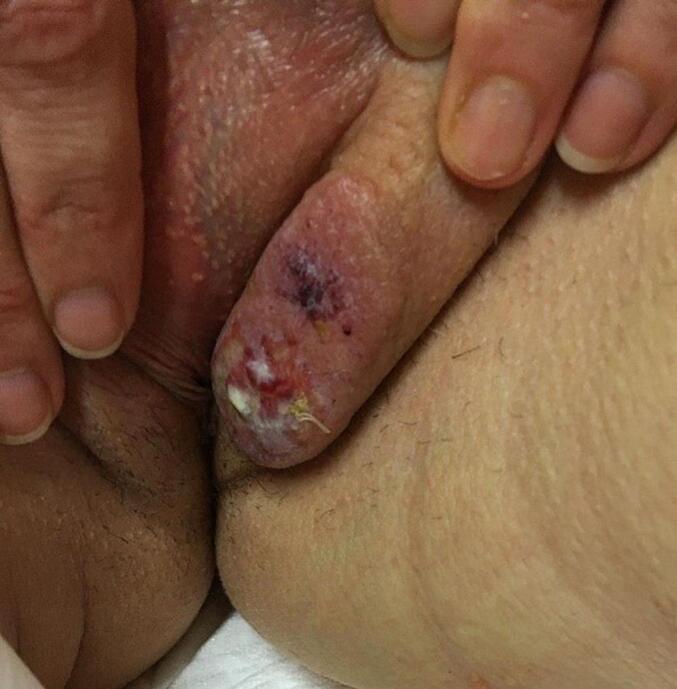
Initial tumor clinical aspect. Primary aspect of the tumor as a pink induration of the left labia major skin, with focal ulceration and whitish deposit. (For interpretation of the references to colour in this figure legend, the reader is referred to the web version of this article.)

The case study reports a 40-year-old nulliparous female who first presented to our institution with a sore and ulcerated induration of the left labia major skin, with whitish deposit and focal lymphoedema (Fig. 1). After months of unsuccessful dermatological treatment for a mislabelled condyloma, diagnosis was made on punch biopsy of the left labia major skin, after careful review of the pathology retaining a low grade epithelioid angiosarcoma, pT1N0M0, stage IA. Neither metastasis nor suspected nodes were detected on the initial PET-CT staging.

**Fig. 2:**
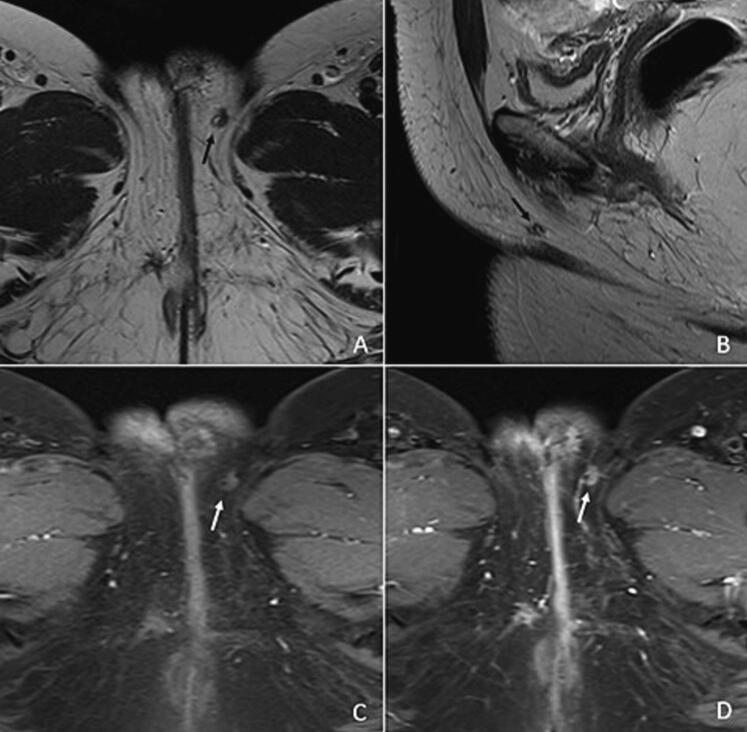
MR image of the superficial tumor recurrence. MR images show a focal well delimitated nodule located in the left labia majora, with a low signal on the T2 weighted-image (A and B), a spontaneous high signal on T1 fat sat weighted sequence (C), and a homogenous enhancement after contrast injection (D).

**Fig. 3:**
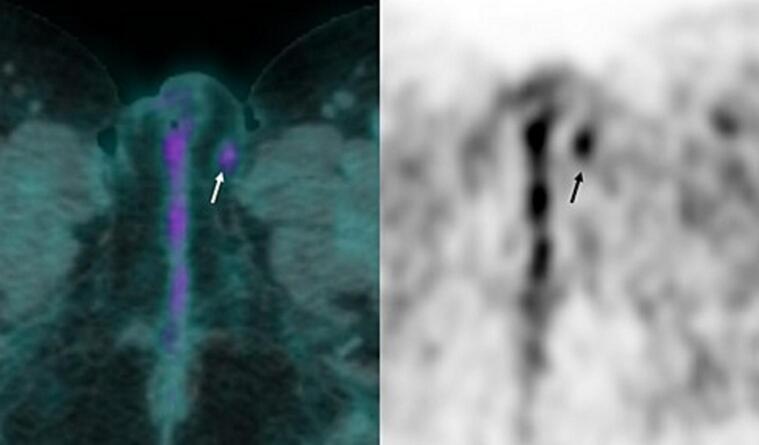
^18^F-FDG PET/CT of tumor recurrence. The PET-CT shows a moderate ^18^F-FDG uptake of the recurrence, with a SUV_max_ value of 2,9 g/mL, compatible with a neoplasm.

A partial left hemi-vulvectomy was performed, with unfortunately positive surgical margins. The patient underwent a new left unilateral vulvectomy and left reconstructive lotus-flap based on the perforator of the internal pudendal artery, with clear margins on pathology. Simple post-surgery imaging follow-up was decided then at tumor board, with no adjuvant therapy. On the MRI and thoraco-abdominopelvic CT at 3 months, an 8 mm mass was suspicious of local recurrence (low signal on the T2 weighted-image, spontaneous high signal on T1 fat sat weighted sequence, and homogenous enhancement after contrast injection) (Fig. 2, Fig. 3). A third left radical vulvectomy was decided.

Due to the particularly small size of the lesion, non-clinically palpable due to the fibrous tissue post-surgery, we decided to radiologically mark the tumor using a magnetic seed.

**Fig. 4:**
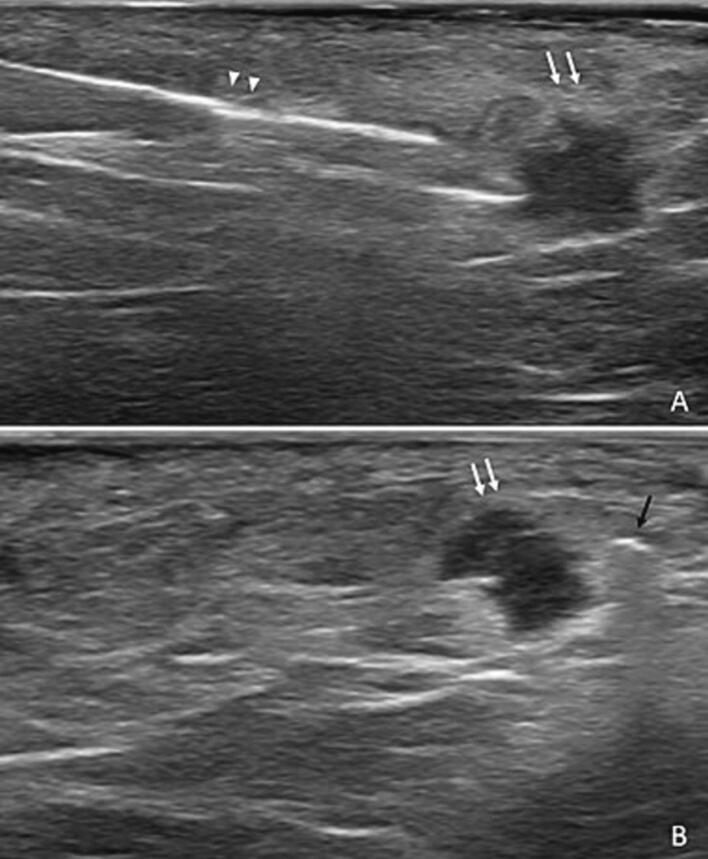
Magnetic seed implantation under ultrasound guidance. After a local disinfection and anesthesia, the magnetic seed (black arrow) was implanted next to the tumoral lesion (white arrows) using a 14G sterile needle (arrowhead), under ultrasound guidance. Neither immediate nor delayed complications occurred.

**Fig. 5:**
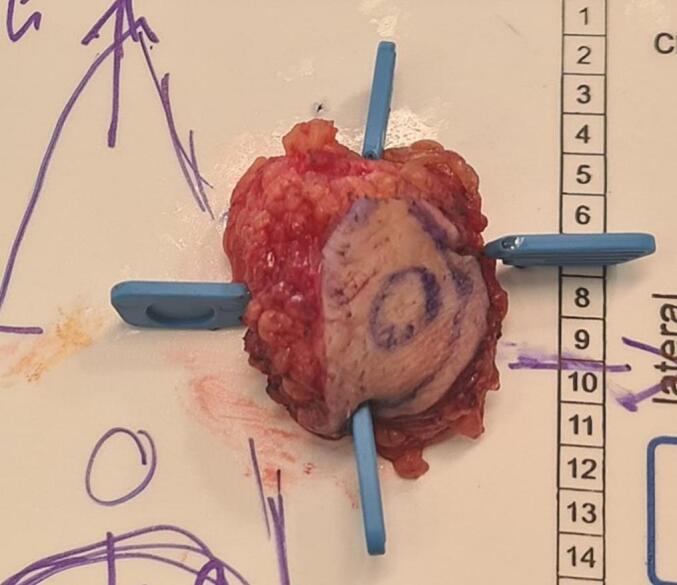
Surgical specimen removed, with the magnetic seed inside. Macroscopically, the tumor was revealed after sections of the removed specimen and appeared as a whitish and indurated lesion.

A magnetic seed was inserted under ultrasound guidance (Fig. 4). Neither immediate nor delayed complications occurred. Surgery was performed 6 days later. Guided by auditory and visual guidance systems provided by the wireless device, we were able to carry out a minimal resection with healthy margins on heavily remodeled tissue (Fig. 5). The final pathology confirmed the healthy margin resection.

At last follow-up PET/CT at 4 months post-surgery, the patient had no clinical or radiological recurrence.

## 3 Discussion

This case presents one of the first applications of magnetic seed localization for tumoral resection technique in vulvar cancer.

The current trend of management of vulvar located cancer is to perform minimal surgical procedures [14]. Many of these lesions are difficult to identify during surgery, either because of the anatomic region or because of the tissular changes induced by neoadjuvant treatment and surgery in case of recurrence. As well as in breast cancer surgery, targeting these tumors with markers helps surgeons to perform a precise and focal removal instead of radical and wide excisions.

Indeed, the more tissue is removed, the greater the risk of the complications [15,16]. Vulvar surgery is known to be at risk of wound breakdown, lymphedema and lymphocele, fibrotic changes with esthetical and functional impacts, local numbness, neuralgia.

Using image-guided localization techniques could help reducing the incidence of these complications, with a minimal excision volume.

Several studies have proven decreased rates of tumor recurrence in patients with breast cancer who beneficiated from image-guided pre-operative localization [17]. By targeting non palpable and superficial lesions, surgeons have greater ability to perform complete removal of the tumor and to optimize the resection margins which remains one of the most important prognostic factor [8,16,18].

Vulvar cancer presents a high overall incidence of local recurrence, all histological types combined. Many of these patients are difficult to examine as part of the follow-up, mainly due to radio-induced changes such as skin induration. Our experience made us assume that image-guided techniques could be helpful in the management of these cases too, optimizing the excision in a scar and fibrotic tissue.

In the present case, due to the localization of the recurrence, the use of a wire was not conceivable. Therefore, the use a magnetic seed to localize the lesion appeared ideal. In addition, and unlike wire devices which must be ideally implanted less than 24–48 h before surgery, magnetic seeds can be inserted up to 30 days before the intervention, without any associated radiation. This facilitates the coordination between radiologists and surgeons, improving scheduling efficiency and patient comfort [7].

As well as for breast cancer surgery, magnetic seed is an easy and feasible system to implant into perineal tumors [19,20]. In the present case, the whole localization procedure took only 35 min, without difficulties or complications.

Moreover, a recent study suggested that magnetic seed devices minimize the volume of removed tissue compared to wire localization techniques in breast cancer (*p* = 0.039) [9]. This could have a significant impact in vulvar surgical procedure, which tends to be as conservative as possible.

The limitations of this technique concern the lesion localization. Indeed, the recommended depth for magnetic seeds implantation and detection is up to 30 mm, limiting the indications to superficial lesions [19].

## 4 Conclusion

In conclusion, with derived use of the image-guided techniques that has been validated in breast cancer, the use of a magnetic seed was successful in a patient suffering from vulvar cancer recurrence. Safe surgical margins and no complications were achieved. Further studies should be performed to assess the efficiency of image-guided localization techniques in superficial gynecological tumors.

## Consent

Informed consent was obtained from the patient for publication of this case report and accompanying images.

## Ethical approval

Patient approval has been given. This study is exempt from ethical approval in our institution.

This article does not contain any studies with human participants or animals performed by any of the authors.

## CRediT authorship contribution statement

Pauline Chapellier:

- study concept or design
- data collection,
- data analysis
- writing the paper

Dr. Basile Pache:

- study concept or design
- data collection,
- data analysis
- writing the paper

Dre Anna Evdokimova:

- study concept or design

Dre Laura Haefliger:

- writing the paper

Dr. Loïc Lelièvre:

- writing the paper
- study concept or design

Pr Jean-Yves Meuwly:

- writing the paper

Pr Patrice Mathevet:

- study concept or design
- writing the paper

Dr. Rami Hajri:

- study concept or design
- data collection,
- data analysis
- writing the paper

## Guarantor

Pr Jean-Yves Meuwly and Dr. Rami Hajri.

## Declaration of competing interest

None.
